# Small Molecules Targeting Biological Clock; A Novel Prospective for Anti-Cancer Drugs

**DOI:** 10.3390/molecules25214937

**Published:** 2020-10-26

**Authors:** Sadia Rahman, Karlo Wittine, Mirela Sedić, Elitza P. Markova-Car

**Affiliations:** Department of Biotechnology, University of Rijeka, 51000 Rijeka, Croatia; sadiahusen@gmail.com (S.R.); karlo.wittine@biotech.uniri.hr (K.W.); msedic@biotech.uniri.hr (M.S.)

**Keywords:** circadian rhythm, biological clock, cancer, small molecules, anticancer drugs

## Abstract

The circadian rhythms are an intrinsic timekeeping system that regulates numerous physiological, biochemical, and behavioral processes at intervals of approximately 24 h. By regulating such processes, the circadian rhythm allows organisms to anticipate and adapt to continuously changing environmental conditions. A growing body of evidence shows that disruptions to the circadian rhythm can lead to various disorders, including cancer. Recently, crucial knowledge has arisen regarding the essential features that underlie the overt circadian rhythm and its influence on physiological outputs. This knowledge suggests that specific small molecules can be utilized to control the circadian rhythm. It has been discovered that these small molecules can regulate circadian-clock-related disorders such as metabolic, cardiovascular, inflammatory, as well as cancer. This review examines the potential use of small molecules for developing new drugs, with emphasis placed on recent progress that has been made regarding the identification of small-molecule clock modulators and their potential use in treating cancer.

## 1. Introduction

The circadian (derived from circa, which means “approximately,” and diem, meaning “day”) rhythms are an internal timekeeping system that evolved in organisms over millions of years and that allows organisms to cope with the daily cycle of light and darkness. The circadian rhythm is commonly referred to as the “body clock” and is recognized as an endogenously driven (approximately) 24-h cycle.

Physiological processes, such as sleep and wakefulness, appetite, hormone levels, and metabolism, are entwined with the effects of circadian rhythms. Hence, these processes are influenced by whether it is day or night. For instance, in humans at nighttime, the rhythms that regulate core temperature, blood pressure, and urinary potassium excretion slow down. Conversely, cortisol and many other endocrine rhythms (e.g., growth hormone and melatonin) tend to peak overnight during sleep. Such rhythms are guided by environmental signals, light-dark (LD) cycles, and food intake. Virtually all organisms have an internal biological clock that regulates their appetite, sleep-wake cycle, endocrine and metabolism regulation, gene expression, and many other fundamental physiological functions [1,2].

The master clock that regulates circadian rhythm comprises approximately 20,000 neurons that form the suprachiasmatic nucleus (SCN). The SCN is located in the hypothalamus, which is the part of the brain that receives direct input from the eyes. Retina absorbs light signals by intrinsically photosensitive retinal ganglion cells (ipRGCs). The ipRGCs are small subset of mammalian RGCs and these cells express an opsin photopigment called melanopsin. Photic signals are transmitted to the SCN via neural pathway called retinohypothalamic tract (RHT) to ensure that the SCN neurons are synchronized with the gradual changes in daylight hours as the seasons change [3]. The SCN clock dictates (either directly or indirectly) daily changes in body temperature, and it controls fluctuations in the levels of several hormones to synchronize the various circadian clocks throughout the brain and peripheral organs [4].

Circadian rhythms are generated cell-autonomously. So-called clock genes (which are essential clock components) form transcriptional regulatory networks within each cell of the body. At the molecular level, the circadian clock system consists primarily of multiple positive and negative transcription-translation autoregulatory feedback loops. These feedback loops cause clock genes to oscillate for about 24 h, thereby producing circadian rhythms via the output system. The core circadian elements involved in regulating a wide range of circadian rhythms are *PERIOD (PER1, PER2* and *PER3*), *CRYPTOCHROME (CRY1* and *CRY2)*, *CLOCK* (circadian locomotor output cycles kaput), *NPAS2* (neuronal PAS domain protein), *BMAL1* (brain and muscle ARNT-like protein 1, referred in nomenclature as ARNTL), retinoic acid-related orphan nuclear receptors *RORα* and *REV-ERBα*, *CASEIN KINASE 1ε (CK1ε)* [2,5,6,7,8].

**Figure 1:**
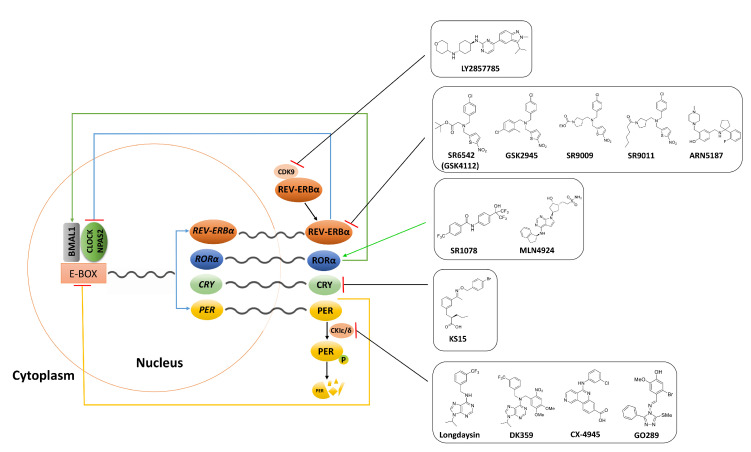
Diagrammatic description of the core circadian clock. The molecular clock is driven by transcriptional-translational feedback loops of the core clock genes and the oscillations of the clock-driven genes are regulated by transcriptional factors CLOCK/NPAS2 and BMAL1. The CLOCK/NPAS2 and BMAL1 heterodimerize and stimulate the expression of *PER* and *CRY* genes as well as *ROR* and *REV-ERB* genes by binding to an E-box region in their promoters. REV-ERB inhibits transcription of CLOCK/NPAS2 whilst ROR promotes Bmal1 expression. Additionally, PER is phosphorylated by CK1*ε* and degraded by proteasomes. Inhibition of CDK9 enhance REV-ERB*α* expression, thereby facilitating the liberation of REV-ERB*α* from CDK9. Molecules that inhibit or activate circadian core clock components in cancer are shown in black boxes.

The entire loop relies on genes that act both as positive regulators such as bHLH-PAS (basic helix-loop-helix-PAS) transcription factors CLOCK/NPAS2, and BMAL1 and negative regulators such as PER and CRY, in oscillators. Regarding positive regulators, BMAL1 and CLOCK/NPAS2 heterodimerize and initiate the transcription of the three *PER* genes and the two *CRY* genes, as well as the *RORα* and *REV-ERBα* genes by binding to an E-box enhancer region in their promoters. As for negative regulators, once PER and CRY proteins reach the critical concentration, they translocate from the cytoplasm and enter the nucleus. From within the nucleus, these proteins inhibit BMAL1-CLOCK/NPAS2 transcriptional activity, thereby restricting their own expression. In the second feedback loop, ROR*α* promote the expression of BMAL1 and CLOCK/NPAS2, while REV-ERB*α* represses the expression of *BMAL1* and *CLOCK/NPAS2*, thus regulating their transcription (Figure 1). Also, PER and CRY are phosphorylated by CK1*ε* and marked for degradation mediated by proteasomes [9]. Furthermore, most clock proteins are translationally changed by the various kinases and phosphatases that are essential to maintaining the circadian rhythm [10].

The circadian clock can be disrupted, and clock genes can be altered by shiftwork, jetlag, and poorly timed food intake, among other factors [11,12]. When this happens, the body’s internal timing regulations can be offset. This situation can cause anomalies in cell proliferation, apoptosis, DNA responses, and metabolism, which, in turn, can contribute to tumor formation and growth. Furthermore, the risk factors for various chronic diseases (e.g., sleep disorders, metabolic syndromes, cardiovascular diseases, affective disorders, neurodegeneration, and tumorigenesis) are known consequences of circadian disruptions. Circadian disruptions are associated with the high incidence rates of various cancers (e.g., lung, breast, ovarian, prostate, pancreatic, endometrial, and colorectal cancer (CRC), as well as hepatocellular carcinoma (HCC), osteosarcoma, acute myeloid leukemia, non-Hodgkin’s lymphoma, and head and squamous cell carcinoma (HNSCC) [13,14,15,16].

As our understanding of the key features underlying the overt circadian rhythm (and its influence on physiological outputs) has continued to expand, it has become apparent that controlling the circadian rhythm via pharmacological means can help to manage circadian clock-related disorders. Many studies have identified several small molecular compounds that can modulate circadian clocks. In this review paper, we discuss small molecules and their potential applications for treating cancer.

## 2. Interrelation of Circadian Clock Genes and Cell Cycle

It is widely believed that there are two regulatory mechanisms (i.e., the circadian cycle and the cell cycle) that affect all biochemical reactions in cells. Therefore, it is rational to conclude that any disturbance to one of these mechanisms will induce the dysregulation of the other and will ultimately have adverse effects on the cell [17]. Enhanced tumorigenesis and the rapid growth of tumors are problems that are frequently associated with dysregulation of the cell cycle [18,19].

The cell cycle is a period of continuous cell growth and DNA replication, followed by cell division. It is believed that most human cancers arise from disturbances in the G1/S cell cycle [20]. The molecular clocks located in peripheral tissues, as well as the core circadian genes, simultaneously exert control over cell proliferation by regulating genes that are associated with the cell cycle. Also, the molecular clocks located in peripheral tissues respond directly to DNA damage and, hence, could be a crucial component of cell cycle control and planned cell death [21].

The circadian clock located at the SCN of the hypothalamus controls the expression of genes associated with the cell cycle. In turn, these genes influence the expression of active Cyclin B1-Cdc2 kinase, which is a fundamental regulator of mitotic cell division; among such genes, *Wee1* expression is known to be directly controlled by circadian clockwork [22]. Research has revealed that the loss of PER2 function in mice caused the overexpression of *c-Myc*. This condition led to DNA damage and, ultimately, the development of hyperplasia and tumors. In other research, *Per2* mutation was found to partially diminish P53-dependent apoptosis, thus increasing the number of damaged cells [23]. Another study showed that *PER1* overexpression induces *c-Myc*. At the same time, it limits the expression of *Wee1*, *CyclinB1*, *CyclinD1*, and *CDK1*, and suppresses p21 in response to ionizing radiation. This activity eventually reduces the proliferation of cancer cells [24,25,26].

## 3. Oncogenesis and Circadian Clock

Many studies have revealed that deregulated circadian clock genes lead to many diseases, including neoplastic transformations. Circadian gene aberrations, such as the mutation, deregulated expression, and even translocation of period genes, have been documented in diverse types of cancer, including breast cancer, prostate cancer, CRC, endometrial cancer, lung cancer, and different types of lymphoma and leukemia as well as HNSCC [13,14,15,16,27,28] Table 1. Several studies have revealed that female workers who frequently have rotating work schedules or who work night shifts are more vulnerable to endometrial and breast cancers. In particular, females who have worked night shifts for more than 20 years may have an increased risk of developing breast cancer [13,29,30].

Other studies have revealed that *PER1*, *PER2, PER3*, and *CRY2* expression are downregulated in breast cancer tissues, whereas *CLOCK* and *TIMELESS* expression are upregulated [31,32]. It has also been suggested that *PER2* is downregulated in HCC, chronic myeloid leukemia, pancreatic cancer, and CRC, HNSCC, and breast cancer but not in endometrial cancer [28,33,34,35,36]. In contrast, *PER2* is upregulated in gastric cancer (GC), as is *CRY1* in the advanced stages of GC [37].

Yu et al. suggested that *CRY1* significantly influences the development and progression of CRC, meaning that it could be a prognostic biomarker of CRC [38]. Another study reported that the expression of *CRY2* is decreased by the progression and prognosis of breast cancer [39]. Moreover, researchers have found that a low level of *NPAS2* expression exacerbates the associations among tumor size, TNM stage, and tumor distance metastasis in colorectal cancer patients [40]. In related work, *NPAS2* was found to function as a potential tumor suppressor gene, and it might serve as a prognostic biological marker for breast cancer and colorectal cancer [40,41].

**Table 1:** Correlation between cancers and circadian clock genes.

Cancer Type	Deregulated Clock Genes	Results	References
Breast Cancer	*PER1*, *PER2*, *PER3*, *CRY2*	Downregulation	[31,32,39]
	*CLOCK*, *TIM*	Upregulation	
Hepatocellular carcinoma (HCC)	*PER2*	Downregulation	[33]
Chronic myeloid leukemia (CML)	*PER2*	Downregulation	[34]
Pancreatic Cancer	*PER2*	Downregulation	[36]
Colorectal cancer (CRC)	*PER2*, *NPAS2*	Downregulation	[40]
Gastric Cancer	*PER2*, *CRY1*	Upregulation	[37]
Head and squamous cell carcinoma (HNSCC)	*PER1*, *PER2*, *PER3*, *BMAL1*, *CRY2*	Downregulation	[28]

Another study indicated that decreased *BMAL1* expression accelerates tumor development and might influence the body’s response to anticancer drugs [42]. In comparison to healthy tissues, malignant HNSCC tissues exhibit lower expressions of *PER1*, *PER2*, *PER3, BMAL1*, and *CRY2*—the lower expressions of *PER1* and *PER3*, in particular, decrease one’s chances of survival [28]. Collectively, these findings emphasize the circadian clock’s significant role in malignant pathogenesis.

## 4. Chemoresistance and the Circadian Clock

Circadian clock proteins have been previously demonstrated as potential biomarkers of treatment response and survival of cancer patients, and their involvement in the development of chemoresistance has been documented in different cancer types, as briefly summarized below.

The expression of *CLOCK* mRNA and protein is greatly increased in cisplatin-resistant ovarian cancer cells, whereas *CLOCK* knockdown significantly increases anti-proliferative and pro-apoptotic effects of cisplatin in resistant cells [43]. Overexpression of CLOCK protein in cisplatin-resistant cancer cells was confirmed in the similar study that also revealed a significant correlation between CLOCK expression and cisplatin sensitivity [44]. Activating transcription factor 4 (ATF4), a member of the cyclic adenosine monophosphate responsive element-binding (CREB) protein family, was found to be a direct target of CLOCK, and downregulation of either CLOCK or ATF4 increased susceptibility of A549 lung cancer cells to cisplatin and etoposide, which suggests that CLOCK and ATF4 transcription system plays an important role in chemosensitivity [44]. Additional evidence to corroborate the role of *CLOCK* gene in cisplatin resistance in ovarian cancer was provided by the study showing that silencing *CLOCK* gene expression reverses cisplatin resistance in ovarian cancer cells by suppressing autophagy and reducing the expression levels of proteins encoded by drug resistance genes P-gp and MRP2 [45]. Altogether, these studies indicate that the *CLOCK* gene may be a novel candidate for targeted therapy in drug-resistant ovarian cancer.

CLOCK forms a complex with BMAL1 to regulate the expression of the *PERIOD* (*PER1*, *PER2*, and *PER3*) and *CRYPTOCHROME (CRY1* and *CRY2*) genes, which in turn act as repressors of their own transcription [46]. Expectedly, suppression of *CRY2* activity could be an effective mean to increase the efficacy of chemotherapy in CRC, as evidenced by the study showing that *CRY2* knockdown increased the sensitivity to oxaliplatin in CRC cell lines. FBXW7, a novel E3 ubiquitin ligase, was identified as the major regulator of CRY2-mediated chemosensitivity, which promotes ubiquitin-mediated degradation of *CRY2* leading to downregulation of *CRY2* and increased CRC cell response to oxaliplatin [46]. Importantly, reduced expression of FBXW7 was found to correlate with high *CRY2* expression in CRC tissue samples and with poor survival, which clearly underlines the importance of FBXW7-CRY2 axis in CRC chemoresistance and puts forward *CRY2* as a novel target to counteract chemoresistance in CRC.

*PER3* was previously demonstrated to play a tumor suppressive role in colorectal cancer [47,48] and its decreased expression has been associated with incidence and progression of colon cancer. In addition, *PER3* is downregulated in colorectal cancer stem-like cells (CSCs) and in drug-resistant colon cancer cells in vitro [49], which clearly indicates that deregulation of *PER3* expression might be an underlying event in the development of chemoresistance in colon cancer. Indeed, an overexpression of *PER3* potentiates the effects of chemotherapy in colorectal CSCs in vitro and reduces their clonogenic and self-renewal capacity concomitant with the inhibition of cancer stem cell-related signaling pathways including Notch-1 and β-catenin, which reduces the chemoresistance and self-renewal capability of colorectal CSCs [49]. Thus, novel agents that could activate *PER3* to suppress cancer stemness could emerge as a promising strategy to reverse chemoresistance in colon cancer.

Similarly, downregulation of *PER2* expression at protein and gene level was implicated in the resistance to cisplatin in lung adenocarcinoma cells [50]. The same study clearly demonstrated that PER2 knockdown promoted proliferation and survival of resistant lung cancer cells by activating the PI3K/AKT/mTOR signaling pathway, whereas overexpression of PER2 protein inhibited this signaling pathway and induced apoptosis of resistant cells. Interestingly, mutation of the *PER2* gene in oncogenic cells was also shown to impart resistance to common chemotherapeutic drugs including methotrexate, gemcitabine, etoposide, vincristine and oxaliplatin by up-regulating the level of the gene encoding for aldehyde dehydrogenase 3a1, whose suppression of cytotoxic effects of anti-cancer drugs could be ascribed to the prevention of reactive oxygen species accumulation [51]. The role of *PER2* in mediating cancer chemoresistance was also demonstrated in pancreatic cancer. Thus, Oda et al. showed that an overexpression of *PER2* had anti-proliferative and pro-apoptotic effects in human pancreatic cancer cells and potentiated cytotoxic effect of cisplatin in a synergistic manner [52].

In contrast to the aforementioned studies, PER2 seems to have an opposite effect on the treatment response in ER breast cancer cells (MDA-MB-231) suggesting cell and genotype-specific circadian control of apoptotic and cell cycle processes [53]. This study shows that PER2 silencing increases the susceptibility of the chemoresistant MDA-MB-231 breast cancer cells to the cytotoxic effects of doxorubicin resulting in S phase arrest and induced apoptosis. Similarly, knocking down *Per1/2* in mice (Per1/2^-/-^mice) increased the efficacy of cisplatin in reducing melanoma tumor growth rate at both low and high doses, and this effect could be attributed to activation of immune response mediated by CD4+ and CD8+ T cell populations, which clearly demonstrates that circadian clock genes, specifically *PER2*, affects the immune response to melanoma tumors [54].

Studies on the role of BMAL1 in orchestrating cancer chemoresistance have provided rather controversial results showing the ability of BMAL1 protein to either protect or sensitize cancer cells to cytotoxic insults induced by different chemotherapy agents in diverse cancer types, which requires further investigation as to assess whether the influence of BMAL1 protein expression on the treatment outcome in cancer is therapy- or tumor type-specific. Thus, an overexpression of BMAL1 in tongue squamous cell carcinoma cells was shown to increase cytotoxic effects of paclitaxel in vitro and in vivo, and its expression levels in tumor tissues from tongue squamous cell carcinoma patients positively correlated with paclitaxel efficiency, which indicates tumor suppressor role of BMAL1 [55]. Similarly, *Bmal1* overexpression increases the response of colorectal cancer to oxaliplatin in vitro and in vivo and its high expression levels are associated with better outcomes in colorectal cancer patients [56]. However, results of this study stand in stark contrast to findings by others. Thus, oncogenic role of *BMAL1* was confirmed in the study showing that *Bmal1* knockout mice is highly sensitive to the anticancer drug cyclophosphamide [57]. Similarly, *BMAL1* knockout increased the sensitivity of non-tumorigenic human breast epithelial MCF10A cells and metastatic human breast cancer MDA-MB-231 cells to cisplatin and doxorubicin while enhancing the invasive capacity of MDA-MB-231 cells, which confirms that disruption of *BMAL1* gene may have opposing carcinogenic effects [58]. Finally, Burgermeister et al. [59] have reported that high protein expression of BMAL1 confers resistance to anti-angiogenic therapy in CRC. These authors have found that high BMAL1 protein expression correlates to non-responsiveness to anti-angiogenic therapy with bevacizumab in mouse models of colorectal cancer and CRC patients and to poor clinical outcome in CRC patients following bevacizumab treatment [59]. Further mechanistic study to better understand observed pre-clinical and clinical findings revealed that BMAL1 upregulates the expression of the vascular endothelial growth factor A (*VEGFA*) gene together with nuclear-receptor-subfamily-1-group-d-member-1 (NR1D1/REV-ERBα) thereby conferring resistance to bevacizumab [59].

## 5. Screening Method for Circadian Clock Modulators

The disruption of circadian rhythms increases the risk of the pathogenesis of various chronic diseases, including cancer [11,12]. Fortunately, researchers have discovered several small molecules that can modulate circadian clocks to treat clock-related disorders, including malignancy. Researchers have identified these kinds of small molecules primarily using two complementary methods: Phenotype-based screening and target-based screening.

Phenotypic screening is employed to measure various circadian parameters, including period, phase, and amplitude. Bmal1 and Per1/2 promoters have been fused to luciferase to allow measurements of the circadian activity of several cell lines that have robust circadian rhythmicity [60]. Researchers have observed circadian transcription or protein oscillation by which stable cell lines expressed either luciferase from an exogenous clock gene promoter or from the fusion of PER2: Luciferase from the exogenous Per2 promoter [61]. Moreover, previous studies have found a small molecule, KL001 through circadian phenotypic screening. This molecule acts specifically upon the central circadian protein CRY, thus prohibiting its ubiquitin-dependent degradation and ultimately prolonging the circadian cycle [62].

In target-based screening, small molecule modifiers can be identified based on their direct interactions with specific clock proteins or regulatory factors. A systematic chemical derivation of small molecule analogs built on prior knowledge of known ligands and/or binding cavity structures must be performed to produce novel and/or enhanced ligands [63]. Previously, this method has been implemented to REV-ERBs and RORs nuclear hormone receptors, which represent the stability loop of the central oscillator. This method has enhanced the development of drugs intended to identified clock proteins via detectable in vitro actions. Although enzymes such as protein kinases can be easily measured, performing in vitro assays for non-enzymatic proteins remain rather difficult [64].

## 6. Management of Clock-Related Diseases Using Small Molecules

As pointed out previously, circadian clock dysfunction is related to several ailments. Several small-molecule compounds that can control various components of the circadian clock have been discovered. These compounds can be utilized to elucidate the molecular activity of the circadian clock and treat clock-related diseases. Previous studies have demonstrated the biochemical impact of REV-ERB agonists such as SR9009 and SR9011 on mice models. When these agonists were used in chronic therapy approaches, they instigated weight loss and decreased body mass due to higher energy expenditure [65].

On the other hand, the administration of an RORγ agonist, SR1555, in diet-induced obese mice reduced body weight by modulating adipocyte function, which led to improved insulin sensitivity [66]. Furthermore, KL001, a CRY activator/stabilizer, prolonged the circadian period and repressed its amplitude. At the same time, it has been found that KL001 impacts hepatocyte glucose synthesis and can be utilized to treat diabetes [61,67]. Jetlag, familial advanced sleep phase syndrome (FASPS), and delayed sleep phase syndrome (DSPS) are some of the most well-known circadian genetic-based sleep disorders. FASPS is characterized by a shortened circadian cycle caused by a mutation in the T44A, CK1δ protein. PF-670462 (a δ-specific inhibitor) indicates that a pharmacological compound can be utilized to prolong the period in patients or animal models with FASPS [61,68]. REV-ERB agonists have also exhibited anxiolytic effects, which is consistent with previous research in which anxiety was increased in REV-ERB*β* knockout mice. Remarkably, the acute administration of SR8278 (a REV-ERB antagonist) in the ventral midbrain of mice also reduced anxiety and promoted manic behavior [61]. Such advanced work in circadian biology has laid the groundwork for improving the treatment of many different clock-related diseases.

## 7. Management of Cancers Using Small Molecules

Little is known about the tumor-intrinsic circadian clock function. Nevertheless, the pharmacological modulation of circadian components might provide selective anticancer strategies. Molecular clock disruption/modulation likely influences the development and progression of cancer [69]. Generally speaking, the circadian rhythm can be targeted in three main ways: (i) By optimizing the circadian lifestyle (“training the clock”), (ii) by optimizing the timing of therapy (“clocking the drugs”), and (iii) by targeting specific circadian clock components (“drugging the clock”) [70].

Recent approaches have attempted to directly target mammalian circadian clock components (i.e., CRYs, REV-ERBs, and RORs) using small molecules. If the entry point is removed too far from the core oscillator, small molecules exhibit pleiotropic effects that do not have to do with their circadian function [61]. Representative synthetic small molecules that influence the circadian core clock in cancer are summarized in Table 2.

## 8. Synthetic Anticancer Chronobiotics

### 8.1. REV-ERBs; RORs; CRY1/2

#### 8.1.1. SR9009/SR9011

REV-ERB*α* and REV-ERB*β* are nuclear receptors and heme-binding circadian clock components that can be activated as a second alternative clock cycle by the CLOCK-BMAL1 complex. They can repress some of the processes involved in tumorigenesis, such as metabolism, proliferation, and inflammation [21,71]. Together with RORs, they form a crucial link between the core circadian oscillator and clock-controlled genes.

REV-ERBs play a key role in lipid metabolism [72], plasma glucose level [73] regulation, and energetic metabolism regulation [74]. Cancer cells have high metabolic demands. As such, it has been postulated that the metabolic pathways of cancer could be altered via the pharmacological modulation of circadian component repressors, such as REV-ERBs, which could compromise the survival of cancer cells [72,73,74]. The first synthetic compound to be identified as an REV-ERB*α* agonist was SR6542 (GSK4112); however, this compound’s pharmacological use is limited due to its unsatisfactory pharmacokinetic profile [75]. The tertiary amine structural motif is common to most GSK4112 analogs and has been questioned, as these types of compounds are known to also act upon LXR*α* (a nuclear receptor that is involved in the regulation of inflammatory and metabolic pathways that overlap with those modulated by REV-ERB) [76,77]. GSK2945 was developed to address this question. In comparison to the starting compound GSK4112, GSK2945 is 10 times more potent and 1000 times more selective for REV-ERB versus LXR*α* [78]. Optimizing the drug-like properties of GSK4112 has led to the development of pyrrole derivatives SR9009 and SR9011.

The findings discussed in the previous paragraph were corroborated by Guido et al. using a glioblastoma multiforme model (T98G cells) and HepG2 cancer cells. The researchers showed that SR9009 acts as a specific agonist against REV-ERBs (a circadian clock repressor), thereby altering tumor metabolism in vivo and exhibiting significant cytotoxic effects. SR9009 also shows cytotoxic effect on brain, leukemia, breast, colon, and melanoma cancer cell lines [79]. Additionally, the researchers assessed cell viability, differential time responses to chronotherapy after synchronization with dexamethasone, and metabolic processes involving ROS and lipid droplet levels. SR9009 treatment significantly reduced cell viability and ROS levels, increased LD levels, had an additive, synergistic effect with bortezomib (a proteasome inhibitor). Approximately 60% of cells treated with SR9009 remained arrested in the G0/G1 phases of the cell cycle [80]. Recently, the in-vitro antitumor effect of SR9009 was confirmed, both in the chemosensitive (H69 and H446) and chemoresistant (H69A and H446DDP) cells of small-cell lung carcinoma (SCLC). SR9009 suppressed the interaction between REV-ERB*α* and Atg5 (a downstream autophagy gene target). Thus, autophagy was impaired at an early stage, which probably restricted the formation of autophagosome. Additionally, SR9009 induced caspase 3-dependent apoptosis, which was partially responsible for the impaired SCLC growth. Additional work confirmed the antitumor effect in a subcutaneous BALB/c nude mice tumor model [81].

#### 8.1.2. SR1078

A synthetic scaffold has been identified that binds and modulates the activity of RORα and RORγ [82]. Based on this, Wang et al. had synthesized an array of compounds and assessed their activity on RORα, RORγ, FXR, LXRα, and LXRβ. Favorable pharmacokinetic properties were confirmed in a mouse model, thus making this compound suitable for in vivo experiments [83]. The down-regulation of RORα was noted in several types of cancer, including breast, ovarian, and prostate cancer [84]. Furthermore, treating HepG2 cancer cells with SR1078 led to p53 stabilization and apoptosis induction [85].

#### 8.1.3. ARN5817

De Mei et al. identified the dual role of ARN5187 as a circadian nuclear receptor (REV-ERBβ) and an autophagy inhibitor [86]. The efficacy of autophagy inhibition as an anticancer strategy has been well documented [87,88]. To withstand starvation and stress, cells consume their own content (e.g., organelles and macromolecules) [89]. The role of autophagy in cancer can be very complex depending on the cellular context, and cell growth can be either promoted or restricted [90,91,92]. ARN5187 disrupts lysosomal function, thereby preventing the complete maturation of autophagosomes, preventing the later stages of autophagy from occurring, and reducing cancer cell viability. The expression of the clock repressor factor REV-ERBβ is higher than that of REV-ERBα in BT-474 cells, as well as in liver (Hep-G2), prostate (LNCaP), and melanoma (A-375 and A-431) cancer cells.

In comparison to the clinically relevant lysosomotropic autophagy inhibitor chloroquine, ARN517 shows significantly higher apoptotic induction activity against breast cancer BT-474 cells. Also, when REV-ERBβ is inhibited, the cytotoxicity of chloroquine is enhanced. Generally, high micromolar concentrations of chloroquine are required to block autophagy in vitro; however, such levels of concentration are rarely achieved [93]. Although experimental data indicate that ARN5187 acts as a cytoprotective factor downstream of autophagy blockades, the exact mechanism by which it functions remains unknown. Overall, the published data support the further development of this scaffold as an anticancer agent [86].

Some further structural modifications have been made to strengthen the activity of this class of compounds. For instance, secondary diphenyl-*N*-cyclopentylamines were identified as effective antagonists of REV-ERBβ [94]. The in vitro cytotoxic activity of the most potent ARN517 compound against a panel of tumor cell lines was superior to that of chloroquine (a well-known autophagy inhibitor) while having a negligible effect on the viability of human mammary epithelial cells.

#### 8.1.4. MLN4924

Pevonedistat (MLN4924) is a small molecule that is structurally related to adenosine-monophosphate (AMP). It inhibits the Nedd8 activating enzyme (NAE) (which is a ubiquitin-like molecule of ~8 kDa, with IC50 of ~5 nM), and it creates a covalent NEDD8-MLN4924 adduct. Currently, it is being evaluated in more than 35 clinical trials related to cancer (www.clinicaltrials.gov). Zhang et al. have shown that MLN4924 stabilizes retinoid orphan nuclear receptor alpha (RORα) by decreasing its ubiquitination and subsequent degradation, which, in turn, transactivates Bmal1 in U2OS osteosarcoma cells [95]. The proliferation of osteosarcoma cells is attenuated by inducing the cell cycle arrest at the G2/M phase and apoptosis. Previously published results reinforce the idea that Bmal1 can transactivate Wee1, which, in turn, might inhibit cyclinB/CDK1 and arrest the cell cycle at the G2/M phase. It has also been demonstrated that MLN4924 can significantly reduce Wee1 activity [96].

#### 8.1.5. KS15

KS15 is a distinctive molecule containing a 2-ethoxypropanoic scaffold and two aryl rings that are connected by an oxime ether linker. This molecule was selected from a pool of more than 1000 compounds known to have drug-like properties. Among the core clock proteins that were tested using a biotin-conjugated KS15 probe, CRY1 and CRY2 were identified as molecular targets, whereas CLOCK, BMAL1, and PERs were not. CRYs are responsible for repressing the clock-controlled gene transcription initiated by the formation of the CLOCK:BMAL1 heterodimer [97,98,99]. Research has shown that KS15 inhibits the repressive function of CRYs and activates the CLOCK-BMAL1-evoked E-box mediated transcription of genes [100]. In contrast to carbazole derivative KL001—which was the first compound shown to directly bind to the core components of the mammalian molecular clock, thus inhibiting both CRY isoforms—KS15 attenuates the circadian rhythm without affecting the period length [67,101].

Moreover, research on MCF-7 human breast cancer cells with functional estrogen receptors confirmed that the inhibition of CRYs promotes E-box mediated transcription. The authors suggest that the elevation of Per2 expression by KS15 is primarily responsible for its antiproliferative and pro-apoptotic activity. Moreover, the chemosensitivity of MCF-7 cells to doxorubicin and estrogen receptor competitive antagonist tamoxifen was increased in other research [102]. Specifically, inhibiting the interaction between CRYs and BMAL1 enhances transcriptional activity. Also, a structure–activity relationship study identified analogs that act upon E-box mediated transcription in a dose-dependent manner that is comparable to the KS15 compound [103].

### 8.2. Casein Kinase

Casein kinase I is a clock regulatory kinase that phosphorylates and degrades PER proteins through a proteasomal pathway, thereby changing the period of the circadian rhythm [104,105]. By screening 1,260 off-patent pharmacologically active compounds, 11 CKI inhibitors of PER2 phosphorylation and subsequent proteasomal degradation were identified (CKI*ε* or CKI*δ*) (IC261, roscovitine, TG003, SB202190, PD169316, SU5416, DRB, SP600125, CGS-15943, PPT, 17-OHP). The period was markedly lengthened in primary cultures of mouse embryonic fibroblasts and human clock cell lines [106].

In other research, the effects of 1,280 different compounds on the length of the circadian period (at a concentration of 7 µM) were analyzed. Out of 13 identified primary hits, 11 were shown to have a dose-dependent influence on cellular circadian rhythms, either lengthening or shortening the circadian cycle. Most of the identified compounds are known anticancer drugs (e.g., roscovitine, SP600125, SB202190, DRB, vincristine, etoposide, mitoxantrone, PMA, SKF-96365, indirubin-3′-oxime, kenpaullone) that exhibit CDK, JNK, and CK2 or microtubule inhibitory activity [107].

#### 8.2.1. Longdaysin

Furthermore, a high-throughput circadian screen of ~120,000 compounds on human cells revealed that longdaysin potently lengthens circadian period in a dose-dependent manner in a variety of mammalian cells from different tissues, including the mouse suprachiasmatic nucleus. The inhibition of CKIδ/ε alone cannot explain the strong modulation properties of longdaysin. Additional protein kinases CKI*α* and ERK2 were also identified as period-regulation targets [108]. The simultaneous inhibition of multiple targets and/or pathways is crucial for its prominent period-lengthening effect. This action is a general characteristic of therapeutically effective anticancer drugs. The period-lengthening effect was also observed for several kinase inhibitors during LOPAC chemical library screening [107]. Notably, the CDK, p38 MAPK, JNK, CK2, and VEGF signaling pathways are often the primary target of compounds with the potential to inhibit CKIδ/ε [106,109,110]. Most high-throughput screening methods found in the literature have identified that period-altering compounds primarily have a period-lengthening capacity. However, circadian amplitude is technically more difficult to quantify [111,112]. The high-throughput screening of 200,000 commercially available synthetic small molecules revealed 11 structurally distinct, independent classes of compounds that display minimal cytotoxicity in fibroblast cells [113].

#### 8.2.2. DK359

Incorporating photocleavable groups (e.g., 2-nitrobenzyl- and nitroveratryloxycarbonyl-(NVOC)) into longdaysin yields derivatives that readily release active molecules upon being illuminated with UV or visible light. In this way, quantitative and light-inducible control over CKI activity was achieved in cultured human cells and mouse tissues, as well as the cells of living zebrafish [114].

#### 8.2.3. CX-4945

An affinity-based proteomics approach designated CK2, which is central to the pathogenesis of cancer, as a target [115,116]. CK2 is a non-oncogene serine/threonine-protein kinase that plays a role in pro-proliferative and anti-apoptotic survival signaling cascades (e.g., PI3K/Akt and WNT), NF-*κ*B transcription, and DNA responses [116,117]. Furthermore, CK2 sustains tumors by supporting the ontogenically-transformed phenotype to protect the cell from increased levels of cellular stress [118]. Moreover, CK2 helps to regulate angiogenesis and HIF-1*α* activity [119,120]. Phosphorylation of PER2 or BMAL1 with CK2 in mammals affects their nuclear accumulation or degradation [121,122,123].

CH-4945 is a phenyl-benzonaphthyridine-carboxylic acid derivative. It is an orally bioavailable low molecular weight compound (MW 350) that exhibits antiproliferative activity against various cancer cell lines. It is a potent and selective ATP-competitive inhibitor of the CK2α and CK2α’ isoforms of the CK2 catalytic subunit (IC_50_ 1 nmol/L). It has also been shown to inhibit DAPK, FLT, TBK, CLK DYRK, HIPK, PIM, and CDK family kinases through the profiling of more than 200 kinases. CX-4945 suppressed angiogenesis (human umbilical vein endothelial cell-HUVEC proliferation, migration, and tube formation) and blocked CK2-dependent HIF-1α transcription in hypoxic cancer cell conditions. The decreased phosphorylation of Akt substrate p21 was demonstrated in murine xenograft models, thus corroborated the CK2 mediated antitumor activity of CX-4945. Finally, research conducted on a panel of breast cancer cell lines revealed that sensitivity to CX-4945 is correlated to the expression of CK2α [124].

#### 8.2.4. GO289

Compared to other kinase inhibitors, the chemical structure of GO289 is quite unusual. It consists of phenyl-1,2,4-triazole, a thiomethyl group attached to triazole, and triazole connected to bromoguaiacol via an imine bond. An SAR study showed that bromoguaiacol and triazole are both necessary for the activity.

Oshima et al. evaluated the effects of GO289 and 23 of its derivatives on circadian rhythms in human U2OS osteosarcoma cells harboring Bmal1-dLuc and Per2-dLuc reporters [125]. Among the evaluated compounds, GO289 stood out as a potential inhibitor of multiple phosphorylation sites on clock proteins. It was also found to lengthen the circadian period significantly. Among the hundreds of kinases that are expressed in U2OS cells, compound G0289 showed a remarkable specificity for CK2, especially when compared to CX-4945, which is also known to inhibit members of the DYRK, HIPK, PIM, and CLK kinase families.

The specificity of G0289 for CK2 is probably the result of its interaction with the hinge region of CK2, which is generally highly conserved among kinases. The planar structure and the limitations of rotational freedom due to the presence of an imine bond are thought to be responsible for shape complementarity between G0289 and CKα.

### 8.3. Cyclin-Dependant Kinase (CDK9)

#### LY2857785

Cyclin-dependent kinases are well-known targets of therapeutic value involved in cancer. Cell cycle progression is strictly controlled by several CKDs and their cyclin partners [126]. Furthermore, CDKs help to repair and transcriptionally regulate DNA [127]. It has been shown that CDK1 partially regulates REV-ERBα stability and that cell cycles and circadian rhythms are a coupled oscillatory mechanism that controls and drives biological processes [128,129]. To track CDKs’ effects on PER2 oscillation, Ou et al. examined the role of 17 commercially available CDK inhibitors using the mPer2Luc mouse embryonic fibroblast (MEF) cell line. LY2857785 (IC_50_ = 11 nM), which is a potent CDK9 inhibitor, was shown to enhance REV-ERBα expression, thereby facilitating the liberation of REV-ERB*α* from CDK9 [130]. Furthermore, when compared to CDK9 (which regulates polymerase-based transcription during the elongation step), CDK7 and CDK8 (which participate in RNA polymerase II transcription at the initiation step) do not appear to influence mPER2-LUC oscillation [126,131]. In general, CDK9 is considered a potential target of tune RORE-mediated transcriptional activity in cancer and other circadian disorders (Table 2).

## 9. Conclusions

Circadian rhythms regulate diverse biological processes, and the circadian clock malfunction in mammals is tightly linked to various pathological states. Indeed, circadian gene aberrations have been recognized in different types of cancers. Involvement of clock genes in the development of chemoresistance has been acknowledged as well. However, to date, little is known about tumor-intrinsic molecular clock functions. Even though research and technological progress have been made in cancer treatment area, the mortality rate is still quite high; therefore, novel approaches for treatment of cancer are urgently needed. High-throughput screening is increasingly applied to create small-molecule libraries and identify compounds that affect basic circadian parameters (e.g., circadian period, amplitude, and phase of rhythmicity) in circadian-synchronized cells. Several small molecules have been identified this way so far. Comprehensive target profiling is of vital importance. Primary hits must be validated and characterized carefully; otherwise, compounds that affect either the phosphorylation, SUMOylation, or stability of CLOCK/BMAL1 will be identified as hits. Phenotypic proteomic profiling, tracking changes in the thermal stability of proteins upon ligand binding, and melting curve analyses have been validated as suitable techniques for identifying the targets of circadian rhythm compounds. Protein kinases are becoming increasingly recognized as valuable targets for clock regulation in cancer. A landscape for circadian modulating compounds showed that a multi-kinase network mainly revolves around CK1, ERKs, CDK2, TNIK, and STK26 [132]. With ever expanding understanding of key features underlying the overt circadian rhythm and its influence on physiological outputs, a pharmacological control over circadian rhythm may yield a novel therapeutic strategy for the prevention and treatment of cancer.

## Figures and Tables

**Table 2:** Representative synthetic and natural small molecules that influence circadian core clock in cancer.

Synthetic Anticancer Chronobiotics					
(A) REV-ERBs; RORs; CRY1/2					
Compound	Chemical Structure	Function	Mechanism	System	Reference
SR6542 (GSK4112)	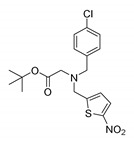	REV-ERB agonist	Inhibition of Bmal1 expression	HepG2 liver cell line	[75]
GSK2945	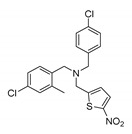	REV-ERB agonist	1000-fold selectivity for REV-ERB versus LXRα	HepG2 liver cell line	[78]
SR9009	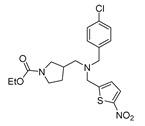	REV-ERB agonist	Circadian clock repressor Cytotoxic properties to a range of cancers e.g., brain, leukemia, breast, colon; in vivo inhibition of in vivo glioblastoma growth	Human gliobalstoma T98G cells; HepG2 cells	[73,79,80,81]
SR9011	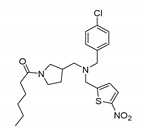	REV-ERB agonist			
SR1078	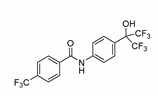	ROR agonist	Activation of target gene expression, such as G6Pase and fibroblast growth factor 21; inhibition of HepG2 and Hep3B hepatoma cell growth both in vitro and in vivo	HepG2 cells and mouse liver	[83]
ARN5187	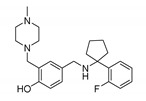	REV-ERBα/β agonist REV-ERBα IC_50_: 790 nM REV-ERBβ IC_50_: 560 nM; Inhibitor of autophagy	Apoptotic induction/anticancer activity; blocking later stages of autophagy	Breast cancer BT-474 cells	[86]
MLN4924	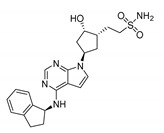	RORα stabilization	Transactivation of Bmal1; induction of G2/M cell cycle arrest and apoptosis	U2OS osteosarcoma cells	[95]
KS15	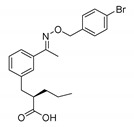	CRY inhibitor	Activation and enhancement of CLOCK-BMAL1-evoked E-box mediated transcription	MCF-7 breast cancer cell line	[100,102,103]
**(B) Casein Kinase**					
Longdaysin	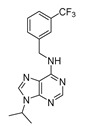	CKIα/δ inhibitor IC_50_: CKIα 5.6 μM IC_50_: CKIδ 8.8 μM	The period-lengthening effect	Cells, tissues, and zebrafish in vivo	[108]
DK359 (caged longdaysin)	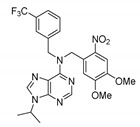	Inactive against CKIα/δ	Temporal control of the clock period	Cells, tissues, and zebrafish in vivo	[114]
CX-4945 (silmitasertib)	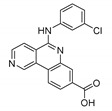	CK2 inhibitor IC_50_: CK2 1 nM	Broad antiproliferative activity; supression of angiogenesis; decreased phosphorylation of Akt substrate p21	Panel of cancer cell lines; human umbilical vein endothelial cell (HUVEC); murine xenograft models	[124]
GO289	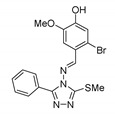	CK2 inhibitor IC_50_: 7 nM	Inhibition of multiple phosphorylation sites on clock proteins	Human U2OS osteosarcoma cells	[125]
**(C) Cyclin Dependent Kinase**					
LY2857785	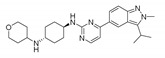	CDK9 inhibitor IC_50_: 11 nM	Enhancement of REV-ERBα expression; liberation of REV-ERBα from CDK9; tuning RORE-mediated transcriptional activity	*mPer2^Luc^* mouse embryonic fibroblast (MEF) cell line	[130]

## Abbreviations

The following abbreviations are used in this manuscript:

LD	Light dark
SCN	Suprachiasmatic nucleus
HNSCC	Head and neck squamous cell carcinoma
CML	Chronic myeloid leukemia
CRC	Colorectal cancer
HCC	Hepatocellular carcinoma
GC	Gastric cancer
